# Atypical Guillain-Barré Syndrome in the Context of COVID-19: A Case Report

**DOI:** 10.7759/cureus.77791

**Published:** 2025-01-21

**Authors:** Cláudia Rodrigues Alves, Patrícia Simões, Bernardo Silva, Jessenia Chinchila, Luís Siopa

**Affiliations:** 1 Internal Medicine Department, Hospital Distrital de Santarém, Santarém, PRT

**Keywords:** atypical presentation, autoimmune polyradiculoneuropathy, covid-19, guillain-barré syndrome, variants

## Abstract

Guillain-Barré syndrome (GBS) is an acute, immune-mediated disease that can be potentially life-threatening. The classic presentation of GBS, characterized by the onset of progressive and ascending muscle weakness following an infectious process, is usually recognized promptly. However, atypical variants with unusual presentations may pose significant diagnostic challenges, resulting in delayed diagnosis and an increased likelihood of unfavorable outcomes. Emerging reports have explored potential associations between severe acute respiratory syndrome coronavirus 2 (SARS-CoV-2) infection and GBS, though the mechanisms underlying these connections remain uncertain. We present the case of a 60-year-old man diagnosed with an atypical form of GBS in the context of COVID-19 infection.

## Introduction

Guillain-Barré syndrome (GBS) is an acute immune-mediated polyradiculoneuropathy and the leading cause of acute neuromuscular paralysis worldwide [1-3]. It is a rare and potentially fatal condition, with an incidence of 0.81 to 1.91 cases per 100,000 person-year [2-4].

The classification of GBS into different variants is based on underlying pathology, clinical presentation, and neurophysiological characteristics [1,2,5]. The classic presentation, known as acute inflammatory demyelinating polyneuropathy (AIDP), is clinically heterogeneous and typically involves progressive and ascending muscle weakness accompanied by diminished or absent deep tendon reflexes, with demyelinating features observed in neurophysiological studies [1,2]. Less common atypical variants include acute motor axonal neuropathy (AMAN), which involves only motor axons due to primary axonal damage, and acute motor-sensory axonal neuropathy (AMSAN), which is similar to AMAN but includes sensory involvement [1,2].

In most cases, GBS is a post-infectious disease, with two-thirds of patients reporting respiratory or gastrointestinal symptoms two weeks before the onset of neurological signs and symptoms [2,3,5]. *Campylobacter jejuni* is the most commonly identified pathogen, leading to GBS in approximately one in every 1,000 cases [3,6].

The diagnosis of GBS is based on clinical history and physical examination, supported by the presence of albuminocytological dissociation in cerebrospinal fluid (CSF) and motor or sensory abnormalities in electrophysiological studies [7]. Treatment primarily involves the administration of intravenous immunoglobulin (IVIg) or plasmapheresis [7].

Severe acute respiratory syndrome coronavirus 2 (SARS-CoV-2)-associated disease (COVID-19) typically presents with respiratory symptoms [5]. However, since the onset of the COVID-19 pandemic, several cases have been reported suggesting a potential association between COVID-19 and GBS, though their pathophysiological correlation remains unclear [4-6,8]. We present the case of a 60-year-old male diagnosed with an atypical form of GBS characterized by an overlap of AIDP and AMSAN variants in the context of COVID-19.

## Case presentation

A 60-year-old man, previously autonomous, with a medical history of undetermined autoinflammatory syndrome diagnosed three years prior, low-risk IgG kappa monoclonal gammopathy of undetermined significance (MGUS), and mitral valvuloplasty due to a myxomatous valve, under regular follow-up in Rheumatology, Hematology, and Cardiology outpatient clinics. Medicated only with prednisolone for a month due to non-specific polyarthralgias. He presented to the emergency department (ED) with complaints of numbness in his hands, which had progressed to the lower limbs and trunk, accompanied by upper limb and gait incoordination. These symptoms began seven days prior and followed a flu-like syndrome with rhinorrhea and dry cough three days earlier. The patient denied other symptoms, including respiratory difficulty, fever, dizziness, palpitations, chest pain, gastrointestinal symptoms, or muscle weakness.

On examination in the ED, he was afebrile, hemodynamically stable with a blood pressure of 143/92 mmHg and a heart rate of 77 bpm, and eupneic on room air with an oxygen saturation of 97%. Neurological examination revealed dysmetria on the finger-nose test on the right side, hypoesthesia of the hands, feet, and trunk, decreased deep tendon reflexes bilaterally, and ataxic gait. Laboratory tests showed no abnormalities, including complete blood count, renal function, electrolytes, glucose level, protein levels, liver function tests, C-reactive protein, procalcitonin, and thyroid function. A urinalysis, chest x-ray, and cranial computed tomography were also unremarkable. Given the flu-like symptoms, Influenza A and B testing and SARS-CoV-2 testing were performed, with a positive result for SARS-CoV-2. A lumbar puncture was also performed, which showed no albuminocytological dissociation or other abnormalities (Table 1).

**Table 1:** Cerebrospinal fluid examination upon admission and after 48 hours.

Examination of cerebrospinal fluid	Admission	After 48 hours	Reference values
Macroscopic examination			
Aspect	Clear	Clear	Clear
Color	Transparent	Transparent	Transparent
Chemical examination			
pH	8.3	8.1	7.2-7.4
Proteins	29.5mg/dL	68.0mg/dL	15-40mg/dL
Glucose	50.0mg/dL	73.0mg/dL	50-80mg/dL
Chlorides	128.9mmol/L	122.3mmol/L	118-132mmol/L
Lactate dehydrogenase (LDH)	<30U/L	34U/L	<40U/L
Microscopic examination			
Total cell count/µL	2 mononuclear cells	Acellular	≤5 cells/µL
Cytomegalovirus (CMV)	Negative	Negative	Negative
Herpes simplex 1,2 (HSV)	Negative	Negative	Negative
Venereal Disease Research Laboratory (VDRL)	Negative	Negative	Negative
Epstein-Barr virus (EBV)	Negative	Negative	Negative

The patient was admitted to the Internal Medicine department for etiological investigation and monitoring. Within the first 48 hours, he developed asymmetric quadriparesis with distal predominance on the right side, hypoesthesia in a high glove-and-stocking distribution and the trunk, bilateral dysmetria, areflexia, and dysdiadochokinesia. Given the worsening clinical presentation, a repeat lumbar puncture was performed, revealing albuminocytological dissociation (Table 1), supporting the diagnosis of probable atypical GBS. Additionally, other causes that could explain the clinical presentation of the patient, such as acute cerebrovascular disease, paraneoplastic syndrome in the context of MGUS, or autoimmune diseases, were excluded. Regarding the etiology of GBS, the etiological investigation identified only a concomitant SARS-CoV-2 infection, with no evidence of infection by other more common pathogens. The patient presented with mild COVID-19, as there was no pneumonia or hypoxemia, and given the progression beyond seven days, no antiviral treatment was initiated.

IVIg was initiated at a dose of 0.4 g/kg. While on IVIg therapy, the patient experienced further clinical deterioration, with worsening motor and sensory deficits, respiratory dysfunction requiring non-invasive ventilation, and dysphagia for solids, necessitating transfer to a level III care unit for close monitoring. The patient completed five days of IVIg treatment and began a motor rehabilitation program, subsequently showing gradual clinical improvement. He was discharged nearly 30 days after the emergency department visit and directed to a rehabilitation unit for continued care.

Although it was requested early during hospitalization, the electromyographic study was only obtained several weeks after hospital discharge. This study revealed widespread abnormalities in motor and sensory conduction studies, consistent with severe mixed sensory-motor polyneuropathy, predominantly sensory, with both demyelinating and axonal involvement (Tables 2, 3). This examination confirmed the diagnosis of an overlap between AIDP and AMSAN, both variants of GBS. The patient achieved complete recovery of deficits approximately one year after the initial diagnosis.

**Table 2:** Motor nerve conduction studies.

	Distal latency (ms)	Amplitude (mV)	Conduction velocity (m/s)	F latency (ms)
Left median nerve				
Wrist-abductor pollicis brevis	8.08 (normal ≤4)	5.6 (normal ≥4)	--	31.2 (normal <30)
Elbow-wrist	13.1	5.3	46.8 (normal ≥50)	--
Right median nerve				
Wrist-abductor pollicis brevis	7.19 (normal ≤4)	1.61 (normal ≥4)	--	33 (normal <30)
Elbow-wrist	12.5	2.6	48 (normal ≥50)	--
Right ulnar nerve				
Wrist-abductor digiti minimi	4.33 (normal ≤3)	4.8 (normal ≥6)	--	33 (normal ≤31)
Left tibial nerve				
Ankle-abductor hallucis brevis	6.47 (normal ≤5.1)	1.39 (normal ≥4)	--	57.8 (normal <52)
Right tibial motor				
Ankle-abductor hallucis brevis	7.13 (normal ≤5.1)	1.17 (normal ≥4)	--	62.8 (normal <52)
Left peroneal nerve				
Ankle-extensor digitorum brevis	8.25 (normal ≤5.5)	2.5 (normal ≥2)	--	--
Right peroneal nerve				
Ankle-extensor digitorum brevis	7.90 (normal ≤5.5)	1.61 (normal ≥2)	--	--
Below fíbula-ankle	18.5	2.3	37.3 (normal ≥42)	--

**Table 3:** Antidromic sensory nerve conduction studies.

	Amplitude (µV)	Conduction velocity (m/s)
Left median nerve		
Digit III - wrist	2.2 (normal ≥18)	36.6 (normal ≥50)
Right median nerve		
Digit III - wrist	4.7 (normal ≥18)	38.0 (normal ≥50)
Left ulnar nerve		
Digit V - wrist	2.2 (normal ≥18)	40.6 (normal ≥50)
Right ulnar nerve		
Digit V - wrist	4 (normal ≥18)	42.4 (normal ≥50)
Left sural nerve		
Mid. lower leg – lateral malleolus	0.84 (normal ≥6)	38.5 (normal ≥40)
Left superficial peroneal nerve		
Calf – medial dorsal cutan.	--	--
Right superficial peroneal nerve		
Calf – medial dorsal cutan.	3.4 (normal ≥6)	41.1 (normal ≥40)

## Discussion

GBS was considered a single entity, thought to result from an immune-mediated attack on myelin components, causing demyelination and subsequent axonal damage [1,2]. However, it was later discovered to encompass a group of autoimmune disorders sharing a common presentation of acute progressive polyradiculoneuropathy, though with different pathogenesis [1,2]. As previously mentioned, the classification into different variants is based on the underlying pathology, clinical presentation, and neurophysiological characteristics [1,2,5]. AIDP is the most common type, corresponding to the typical GBS, with a presentation of progressive, symmetric, and ascending muscle weakness associated with diminished or absent osteotendinous reflexes, which may be accompanied by sensory loss or involvement of the cranial nerves with dysphagia and respiratory difficulty [1,2]. It is also characterized by the presence of demyelination on neurophysiological studies [1,2]. AMAN is less common, primarily presenting with axonal damage and having only motor involvement clinically [1,2]. AMSAN is similar to AMAN but with additional sensory involvement and a worse prognosis [1,2]. Miller-Fisher syndrome (MFS) presents with the classic triad of ophthalmoplegia, areflexia, and ataxia [1,2,4]. Bickerstaff brainstem encephalitis (BBE) is similar to MFS but includes impaired consciousness due to immune attack extending to the pontine reticular formation [1].

The diagnosis of GBS is based on the clinical history and physical examination, supported by CSF analysis, which typically reveals albuminocytological dissociation with elevated protein levels and absence of cells, as well as electrophysiological studies demonstrating reduced motor or sensory conduction [7]. The National Institute of Neurological Disorders and Stroke (NINDS) criteria and the Brighton criteria are the most commonly used diagnostic tools [2,7]. However, many patients present with variable symptoms that do not meet any specific set of criteria. Thus, recognizing all GBS variants is clinically important to avoid diagnostic delays [2]. Most patients recover fully with early diagnosis, but 3%-10% may die during the acute phase [5].

Since the first reported case suggesting a possible association between COVID-19 and GBS in January 2020 in China, more than 200 cases have been documented worldwide [5,6,9]. A 2021 meta-analysis estimated the prevalence of GBS among COVID-19 patients to be 15 cases per 100,000 person-years [5]. Nevertheless, the correlation between COVID-19 and GBS remains controversial [4-6,10].

It is believed that the coronavirus causes GBS as an autoimmune response triggered by the cytokine storm released during the inflammatory response associated with COVID-19 [5,6,8,11]. Case reports so far suggest that COVID-19-related GBS differs from the typical post-infectious pattern and more frequently presents as an acute parainfection, meaning that the patient develops neurological symptoms shortly after the onset of SARS-CoV-2 infection rather than weeks following the infectious process [6,9,11,12]. This difference may be explained by the presence of the virus in the CSF in non-SARS-CoV-2-associated GBS cases, leading to direct damage of nerve roots, a phenomenon not observed with SARS-CoV-2 infections [9,11,12].

Analysis of reported COVID-19-related GBS cases revealed that clinical manifestations and electrophysiological findings are similar to those in GBS caused by other factors. Limb weakness is the most common symptom, with AIDP being the most frequent variant, followed by AMAN [5,6,8,12]. However, cranial nerve involvement appears more common in COVID-19-associated GBS [12]. Additionally, overlapping features of AIDP and AMSAN are rarely reported in such cases [6,8,11]. The prognosis of COVID-19-related GBS appears worse, potentially due to the systemic damage caused by COVID-19, though this does not seem to translate into higher morbidity or mortality rates [5,12].

The clinical case presented here posed a diagnostic challenge due to the atypical initial presentation of GBS, namely sensory impairment, dysmetria, and gait ataxia, but without muscular weakness. The possibility of GBS seemed even more remote given the absence of albuminocytological dissociation in the CSF on admission and due to the inability to perform early electrophysiological studies. Another confounding factor was the patient's prior diagnosis of MGUS, which raised the possibility of a paraneoplastic syndrome, later excluded. Furthermore, the patient had no prior infection with common etiological agents of GBS, and the available literature at the time on the association between COVID-19 and GBS was scarce and presented a clinical picture different from that of our patient. All these factors resulted in a delayed initiation of targeted therapy with IVIg, leading to a significant worsening of the patient's symptoms.

## Conclusions

We report another case of COVID-19-related GBS, contributing to the ongoing discussion about the role of SARS-CoV-2 as a potential trigger for this condition. The presented case underscores the importance of identifying atypical GBS variants, enabling early diagnosis and increasing the chances of full recovery. Despite existing case reports, significant gaps remain in understanding the correlation between these two entities, and the full spectrum of clinical features of COVID-19-associated GBS is still unknown.
